# The Rise and Fall of the Mediterranean Diet and Related Nutrients in Preventing Diabetes

**DOI:** 10.3390/nu14020379

**Published:** 2022-01-17

**Authors:** Maria Mirabelli, Antonio Brunetti

**Affiliations:** Department of Health Sciences, University of Magna Græcia, 88100 Catanzaro, Italy; maria.mirabelli@unicz.it

Four years after the successful narrative review of Alkhatib et al. [1], an online search on PubMed using keywords “diabetes” and “functional food” reveals over 2700 studies on the subject: namely 1300 more than in the previous output [1]. As retrieved from the literature, the best known source of (natural) functional foods is the Mediterranean Diet (MedDiet), which is rich in fresh fruits, vegetables, olive oils, fish, poultry, eggs, herbs and nuts and has traditionally been eaten by people from the lands surrounding the Mediterranean Sea, who were found to be blessed with exceptional longevity from the 1960s to the early 2000s [2]. While pioneering observational/epidemiological works failed to provide evidence of causality in the beneficial relationship between adherence to the MedDiet and healthy aging [3], a subsequent long series of clinical randomized trials and post hoc analyses from the landmark PREvención con DIeta MEDiterránea (PREDIMED) study program, conducted in Spain between 2003–2010, conclusively revealed that adults eating non-calorie-restricted MedDiet recipes, enriched with extra virgin olive oil (EVOO) or nuts, achieve half the risk of developing type 2 diabetes (T2D), cancer or cardiovascular disease during a 4-year period of follow-up with respect to those adhering to a typical low-fat diet [4,5,6].

However, it is important to recall that the MedDiet components are not exclusive to the Mediterranean basin [1], and implementation of the MedDiet outside of this geographical area (i.e., past the “Pillars of Hercules”) is not only possible [7] but also effective in preventing diabetes and future cardiometabolic risk in non-Mediterranean populations. In this regard, it has been recently reported that, in 25,317 initially healthy U.S. women, a higher baseline adherence to the MedDiet was significantly associated with 30% lower risk of T2D during a 20-year period of follow-up [8]. Improvements in biomarkers of insulin resistance (IR) made the largest contribution to the long-term reduction in T2D risk mediated by the MedDiet, followed by changes in BMI, HDL measures, and inflammatory indices [8]. Although the underlying mechanisms still need to be addressed, the beneficial effects of the MedDiet on several IR biomarkers and diabetes outcomes have been extensively reviewed by our own group and others in a dedicated Special Issue of *Nutrients* [9,10]. EVOO has consistently emerged as the principal functional component of the MedDiet, and through its high total polyphenol content and balanced proportions of mono- and poly-unsaturated fatty acids, it has been found to be profoundly involved in the upstream and downstream networks of insulin receptor (INSR) signaling [9]. However, it has to be mentioned again that even the most abundant dietary polyphenols are poorly absorbed by the small intestine and/or rapidly metabolized in humans, with plasma concentrations rarely exceeding 1 µM following natural food ingestion [1]. Only a small number of polyphenols from EVOO and other functional components of the MedDiet are considered to be bioavailable or bioactive within insulin-sensitive tissues and therefore of potential therapeutic value for diabetes prevention [1]. Oleacein, a natural secoiridoid derived from the metabolism of oleuropein, might stand out as a good exception to this rule [11,12].

Compared with other phenolic compounds in EVOO, oleacein possesses a relatively high lipophilicity, which may explain its improved survival to gastric acids and enhanced intestinal absorption in systemic circulation, where it would reach concentrations (up to 18 µM) capable of causing clinically relevant insulin-sensitizing actions, eventually useful for diabetes prevention in people adhering to the MedDiet [11]. Furthermore, the chemical semi-synthesis of oleacein starting from oleuropein (the most abundant and easily accessible constituent of olive leaf extracts) is sustainable, and we should expect that it will be used to enrich commercial oils of beneficial nutritional properties for human health in the near future [13].

Over the last few years, several arguments have been given to the questions raised by Alkhatib et al. [1] regarding the potential combined effect of exercise with functional food consumption and the role of nutrient metabolites as predictors of response to lifestyle interventions. For example, very recently, it has been evidenced that a 12-week intervention with a non-calorie-restricted paleolithic type of diet (emphasizing ad libitum consumption of fresh fruits, vegetables, eggs, fish and lean meats, without added sugars—which is similar to the MedDiet scheme) significantly improved hepatic insulin sensitivity in obese adults with T2D and was associated with reduced ectopic lipids in liver and muscle tissues, along with decreased circulating levels of branched-chain amino acid (BCAA) metabolites, specifically valine [14]. The addition of supervised exercise training, combining aerobic exercise and resistance training (3 h per week) to this type of diet in a matched group of patients did not produce significant changes in either hepatic insulin sensitivity or ectopic lipid accumulation. Instead, an increase was observed in specific lipids, such as diacylglycerols and triacylglycerols, in skeletal muscle, along with an increased fat oxidation capacity and mitochondrial content [14]. This altered pattern of lipids in skeletal muscle has been hypothesized to reflect an increase in lipid utilization and a redistribution of fat towards bioenergetic organelles instead of sites that may perturb INSR signaling (e.g., the plasma membrane).

As shown in the interventional Diabetes Prevention Program study, supervised exercise of moderate intensity (150 min per week) improved clinical outcomes in patients with IR and prediabetes, reducing the chances of developing overt T2D when combined with a typical low-fat, calorie-restricted diet (58% reduction of T2D risk at 3 years [15]; 27% reduction of T2D risk at 15 years [16]; 25% reduction of T2D risk at 22 years [17]). However, despite strong evidence of the potential impact of regular exercise in preventing T2D, shortfalls with exercise prescription are evident, especially in the primary care setting and/or countries where cultural barriers restrict female participation in outdoor activities [18]. Today, the scientific community is actively searching for strategies that could address many of these shortfalls and barriers by investigating the impact of different doses and intensities of exercise on peripheral insulin sensitivity. Much attention is given to high-intensity interval training (HIIT), which appears more feasible (i.e., it can be practiced at home), well tolerated, enjoyable and time-efficient with respect to standard moderate-intensity exercise recommendations for people with or at risk of T2D [19]. Furthermore, in women of reproductive age with polycystic ovary syndrome (PCOS), a persistent IR state associated with strong lifetime risk of adverse cardiometabolic outcomes, including gestational diabetes and T2D [20], there is preliminary meta-analytic evidence that HIIT could be more effective than standard moderate-intensity exercise in improving insulin sensitivity and cardiorespiratory fitness, at least in the short term (within 16 weeks) [21]. Even in this circumstance, the insulin-sensitizing effect of exercise may be linked to the enhanced translocation of glucose transporters to the plasma membrane, in addition to the metabolic and hormonal adaptations that ameliorate INSR expression and function at the skeletal muscle level [21]. Overall, the results of the meta-analysis by Santos et al. [21] support the potential application of HIIT as a valid prevention strategy for T2D in at-risk women with PCOS.

The effects of regular physical activity and functional foods of the MedDiet have often been investigated as separate components, and there is still limited knowledge about their potential synergism in achieving better health outcomes [1]. Partially filling this gap, it has been prospectively documented that adherence to the MedDiet, combined with engagement in high levels of physical activity, had multiplicative effects on all-cause mortality risk reduction in a Spanish population [22], in which incident deaths were related to cancer and cardiovascular events [22], two conditions frequently found in patients with IR and T2D [23]. Even if universally appreciated as a healthy and sustainable nutritional model to prevent a variety of chronic diseases and premature deaths (http://en.unescomeddiet.com/ (accessed on 31 December 2021)), there is still need of more evidence-based public health policies to get the MedDiet adequately adopted worldwide and transmitted to future generations.

In addition to the search for strategies aimed at increasing adherence to the MedDiet in the general population, including people living in Mediterranean countries, it is important to clarify its role under special circumstances, such as in gravid women, when a transitory IR state and complex metabolic adaptations physiologically occur in order to satisfy the nutritional needs of rapidly growing fetus during a healthy pregnancy [24]. It has been estimated that, globally, one pregnancy out of seven is complicated by gestational diabetes, and, in such instances, the maternal metabolic adaptations of pregnancy are led to the extreme [24]. To reduce the risk of perinatal morbidities in infants born to mothers with gestational diabetes, immediately after diagnosis of maternal diabetes, the international guidelines recommend initiating a personalized medical nutritional plan with physical activity, aiming for more stringent glycemic targets and modest gestational weight gain (as per the 2009 Institute of Medicine recommendations) [24]. Although not specifically recommended by guidance authorities, the adoption of the MedDiet for prevention or treating gestational diabetes is expected to lead to positive results. A recent prospective interventional study has evidenced that adoption of the MedDiet during early gestation (i.e., at the end of first trimester), supplemented with EVOO and nuts, significantly reduced (up to 30%) the incidence of gestational diabetes and other adverse pregnancy outcomes, including the risk of excessive gestational weight gain and the need for adjunctive insulin therapy in pregnant diabetic women [25]. Further research is warranted to understand the role of MedDiet and exercise in the modulation of fetal growth and the long term implications for metabolic health in mothers and newborns.

As already stated before [9,10], adherence to the MedDiet affords sustained protection against T2D and other IR states. Besides searching for mechanistic explanations and the identification of specific nutrients from functional foods that may have an impact on glucose homeostasis and peripheral insulin sensitivity, or searching for novel biomarkers of response to dietary interventions [1], we also need to focus on interactions between nutrition and exercise and their potential synergistic effects to fully optimize metabolic health in people prone to develop diabetes and other metabolic diseases.

## Data Availability

Not applicable.
